# The crewed journey to Mars and its implications for the human microbiome

**DOI:** 10.1186/s40168-021-01222-7

**Published:** 2022-02-07

**Authors:** Torben Kuehnast, Carmel Abbott, Manuela R. Pausan, David A. Pearce, Christine Moissl-Eichinger, Alexander Mahnert

**Affiliations:** 1 Institute of Hygiene, Microbiology and Environmental Medicine, Medical University of Graz, Neue Stiftingtalstraße 6, 8010 Graz, Austria; 2 Department of Applied Sciences, Faculty of Health and Life Sciences, Northumbria University at Newcastle, Northumberland Road, Newcastle-upon-Tyne, NE1 8ST UK; 3 BioTechMed, Graz, Austria

**Keywords:** Space, Spacecraft, Space traveler, Astronauts, Healthy, Perturbation, Reconstitution, Diversity, Gut, Planetary protection

## Abstract

A human spaceflight to Mars is scheduled for the next decade. In preparation for this unmatched endeavor, a plethora of challenges must be faced prior to the actual journey to Mars. Mission success will depend on the health of its crew and its working capacity. Hence, the journey to Mars will also depend on the microbiome and its far-reaching effects on individual crew health, the spaceship’s integrity, and food supply. As human beings rely on their microbiome, these microbes are essential and should be managed to ensure their beneficial effects outweigh potential risks. In this commentary, we focus on the current state of knowledge regarding a healthy (gut) microbiome of space travelers based on research from the International Space Station and simulation experiments on Earth. We further indicate essential knowledge gaps of microbial conditions during long-term space missions in isolated confined space habitats or outposts and give detailed recommendations for microbial monitoring during pre-flight, in-flight, and post-flight. Finally, the conclusion outlines open questions and aspects of space traveler’s health beyond the scope of this commentary.

Video Abstract

**Supplementary Information:**

The online version contains supplementary material available at 10.1186/s40168-021-01222-7.

## Background

After landing on the Moon and maintaining crewed stations in low Earth orbit (LEO), such as the International Space Station (ISS), humankind is on the verge of leaving terrestrial boundaries and becoming a space-traveling species. The next step and crucial challenge are setting foot on another planet, such as Mars. Our path to the red planet serves as a first step towards becoming a multi-planetary species and for deeper space missions in the future.

The crew of a human mission would carry their microbiome with them. Quickly after birth, humans are immersed in a community of billions of microorganisms (bacteria, archaea, fungi, protozoa, viruses/phage) thriving on their bodies and in both their natural and built environments. These dynamically exchanging microbiomes are inevitably interwoven with human health and disease. To date, there is a very limited understanding of how space travel may affect microbial biology or even pathogenicity. For example, if exchange with the diversity on Earth is restricted or cut off completely, what affect does this have on the fine balance of microbial influx, equilibrium, and efflux and consequently to human health?

Astronauts encounter a range of conditions that may impact microbiome health, such as social isolation from the general population and extensive hygiene protocols. Somewhat similar limitations have been observed on a much wider scale during the COVID-19 pandemic, with social isolation almost globally used as a control measure to prevent the spread of the disease. These measures possibly impair microbiome diversity on a broad scale [1]. Furthermore, deciphering the effects of a reduced microbial influx on microbial diversity and human health may be key for handling both the pandemic and crewed long-term space missions.

This commentary will first provide important background information on the definition of a healthy human gastrointestinal microbiome and then summarize the current state of knowledge derived from human microbiome analysis in space-relevant settings, including Earth-based simulation experiments and the ISS. Important knowledge gaps will be addressed, and recommendations for future space missions will be made.

## Main text

### A healthy human microbiome—a prerequisite for human space travel

The definition of a healthy microbiome is still subject to scientific debate. Initially, it was hypothesized that a set of core commensal bacteria facilitates a generally healthy state for all individuals. With the emergence of high-throughput sequencing techniques, the consensus has shifted in light of the evidence of a large variety of species found on each person [2]. In particular, current data suggests that the microbiome develops during childhood and establishes immunological tolerance for randomly encountered commensals during the first three years of life [3]. The tolerance for some species is hypothesized to be mediated by an immune mechanism reacting to *short-chain fatty acids* (SCFAs—such as propionate, acetate, and butyrate) produced by commensal bacteria [4]. The role of SCFAs on the host is manifold, including serving as a source of energy, regulation of metabolism in general, and regulation of the immune system [5].

The random set of microbes each individual infant ingests over time is hypothesized to become the individual’s core set of tolerated commensals for years or a lifetime [6, 7]. Generally, at least 160 dominant species can be found in each individual, mainly belonging to the Firmicutes and Bacteroidetes [8–13]. However, the relative abundance of these dominant phyla varies between individuals, and they are usually accompanied by a variety of other taxa.

Under constant conditions, the microbiome can reach an equilibrium of its microbial communities. Therein, the microbiome reaches a stable steady state of species, represented by a weakly fluctuating baseline composition [2, 3]. Depending on the level of diversity, the microbiome possesses sufficient resilience to maintain its *steady state* against perturbations such as diarrhea, antibiotics, laxatives, immunological host reactions, or other external intrusions of species or substances. Species within a stable and highly diverse microbiome seem less likely to be replaced during perturbation. Any given state of the microbiome, including a steady state of high diversity, may be beneficial, neutral, or detrimental to health depending on the composition of the species. Some microbial species may act as commensal bacteria, while more could be potentially capable of pathogenicity but yet inactive (e.g., MRSA) [2, 3]. If a specific equilibrium of the microbiome is linked to detrimental effects to the human host, it is considered to be in *dysbiosis* [3, 14]. Dysbiosis has been shown to date, to be associated with numerous diseases, such as inflammatory bowel disease, urinary stone disease, multiple sclerosis, diabetes, obesity, cardiovascular diseases, allergies, asthma, autism, kidney diseases, and cancer [14, 15]. Microbiomes in dysbiosis are often marked with reduced diversity, i.e., an infection by *Salmonella*, followed by diarrhea and a drop in abundance of regularly dominant species. However, even dysbiotic microbiomes can be diverse and as resistant as healthy ones to perturbations when pathogens manage to settle and induce chronic inflammation [3, 16]. To link these observations to clear causal and mechanistic connections, highly resolved taxonomic, functional, and multiomics analyses are required. In particular, approaching the human microbiome from a functional viewpoint may give a better insight into how to define a healthy microbiome rather than simpler measures such as richness, diversity, and evenness of phylogenetic taxa.

*Perturbations* arise from factors including the introduction of infectious species, diet changes, stress, or antibiotic use, where microbial niches are partially or completely cleansed of the original species depending on the intensity [10, 17]. Once the niche is available, successors can populate it through the process of *reconstitution*. Factors like microbial fitness under the respective niche conditions or tolerance of the immune system towards specific species influence whether the new species is transient or becomes a permanent resident. The stronger the perturbation, the more niches are cleansed and subject to reconstitution either by the original species or by new ones. Consequently, the steady state persists or changes, the latter with unknown implications for the individual’s health status [2, 14]. The re-assortment of old and new microorganisms can either restore the old, healthy steady state; create a new, healthy steady state with some variation from the initial state, or lead to a dysbiotic state [2]. Reconstitution is still a highly diverse and unexplored process and currently subject to intensive research effort.

Taken together, each human gut microbiome comprises an individual set of microorganisms, which under healthy conditions should be constituted by a large variety of commensal and beneficial microorganisms. Optimally, the microbiota produces essential and beneficial substances, arranges itself in a stable equilibrium, occupies the ecological niches of the gastrointestinal tract, and blocks them from pathogenic intruders. If a perturbation destabilizes the steady state, reconstitution should aim to restore the original state.

Unfortunately, with so many yet unknown factors contributing to a healthy or dysbiotic human gut microbiome on Earth, in space, the additional variables such as zero gravity, cosmic radiation, or long-term isolation increase the complexity of these studies. To elucidate a few of these uncertainties, preliminary studies have been performed on Earth and on the ISS, which are discussed in the following.

### Insights from ground-based simulation studies

On a space mission, the crew will encounter all kinds of alterations compared to a regular life on Earth, such as microgravity [18], increased cosmic radiation [19], hermetically sealed isolation, food limitations [20], space-motion sickness [21], stress [22], and many other influencing factors. A mission to Mars can take up to years without the chance to abort, and therefore, the crew has to be well prepared for any problems that arise. Hence, simulations have been conducted including Mars500 and HI-SEAS which are discussed in the following.

The *Mars500* project was an Earth-based experiment simulating an entire mission to Mars between June 2010 and November 2011. Six crew members were confined in an environment resembling a mock spacecraft for the duration of 520 days, the possible length of a crewed Mars mission. Microbiome samples of the crews’ stool and mock spacecraft surfaces were frequently taken and sequences of the 16S rRNA gene were analyzed for species diversity [11, 23, 24]. Measuring beta-diversity (inter-sample diversity, the similarity, or dissimilarity between two communities) of the crews’ microbiome, the median unweighted UniFrac distance dropped by up to nine percent after seven months, while the weighted UniFrac distance followed random fluctuations [11]. These findings could indicate that dominant species kept their prevalence throughout isolation, whereas the less dominant species became more similar. The alpha-diversity (intra-sample diversity) was highly dynamic, and changes in alpha-diversity could not be correlated with external events. After the confinement of the Mars500 experiment, the microbiomes of two subjects returned to the pre-mission baseline, while the other four kept their Mars500 altered diversity for up to six months [11]. These observations substantiate the highly personalized traits of the human microbiome in confined habitats.

The surface microbiome of the Mars500 built environment was found to be mainly composed of taxa assigned to *Staphylococcus* and *Bacillus* [24]. The data suggest that the environment is consistently inoculated by microbes from the individual crew members’ microbiomes. In order to analyze the surface microbiome, three different methods were used in parallel: classical microbial cultivation, PhyloChip analysis, and NGS-based amplicon profiling. Although all were shown to have their advantages, amplicon profiling was able to identify the highest number of taxa [24].

Although quite a number of different analyses (e.g., ethological, psychophysiological, time effects, brain function) were performed during the Mars500 simulation, the interpretation of experimental results could benefit if they had been conducted in a concerted manner (e.g., with respect to the time point of analysis).

Within the *Hawaii Space Exploration Analog and Simulation IV* (*HI-SEAS IV*) mission, microbial transfer between a Martian outpost mock-up habitat (an 11 meter in diameter spherical-shaped dome located at the barren slopes of the Mauna Loa volcano) and the skin of its isolated crew (six crew members) for an entire year was monitored in detail [25]. Despite its confined setup, which included limited hygiene regimes for the crew members, a dehydrated food-based diet, and leaving the habitat only with spacesuit mock-ups, the microbiome was still highly dynamic. These microbial dynamics were driven by ~ 15 specific indicator taxa from either skin, GIT, or the environment. It was possible to use patterns in microbial diversity to trace the interaction of crew members both with the habitat and with each other. In contrast to stool samples from the Mars500 project, microbial diversity of skin samples from the HI-SEAS IV crew successively increased and showed a delayed longitudinal homogenization with microbial diversity estimates from habitat surfaces, mainly due to the complications with the composting toilet. Hence, the authors concluded that the microbiome on the crew’s skin was influenced to a higher extent by the microbiome of the habitat’s surfaces in comparison with common non-confined indoor built environments. Additionally, the observed loss of microbial diversity in stool samples (e.g., Mars500) mainly resulted from the specific and restricted diet.

Mars500 and HI-SEAS IV are exemplars of a number of simulated ground-based missions, which included microbiome analyses, such as the inflatable lunar/Mars analog habitat (ILMAH) [26], the Green Star 180 project [9], Lunar Palace 1 [27], and more. However, conflicting observations across these studies have impaired a coherent understanding of this very complicated situation to date.

### Microbiome analyses of human space travelers

The *International Space Station* (ISS) orbits Earth at around 400 km distance and provides microgravity and interplanetary vacuum, mirroring important traits of interplanetary travel. The station lacks direct contact and exchange with the Earth’s microbiome but receives microbial influx from cargo flights approximately every three months and new crew members approximately every six months.

In general, longitudinal microbiome analyses of crew members of the ISS are extraordinarily rare, but an initial study, involving the analysis of the gut microbiome before, during, and after travel, was published recently [28]. Similar to the Mars500 experiment on Earth, the gut microbial community in four out of five astronauts became more similar during their six-month mission. At the same time, the Shannon alpha-diversity and richness of the individuals’ gut microbiome in space significantly increased (*P* < 0.05 for both measures). These adaptations appeared in the first samples taken on the ISS seven days after arrival and persisted until the end of the mission. After returning to Earth, the median and distribution of the alpha-diversity indices returned to values similar to the preflight baseline over the first two months. The skin alpha-diversity and richness significantly increased for five of nine astronauts and had a non-significant downwards trend for the other crew members [28]. The data showed no detectable loss of species over time under these specific conditions for a presence of at least six months on the ISS. These observations suggest resilient capacities of the human microbiome even after exposure to space conditions.

In a 340-day mission, the gut microbiome of monozygotic *twins* (both astronauts) was monitored by shotgun metagenome sequencing, with one twin residing on the ISS and the other remaining on Earth [29]. Overall, the richness and Shannon index of the twin on the ISS did not change significantly (comparing inflight samples to pre- and post-flight samples). However, the composition of the inflight microbiota samples significantly changed compared to pre- and post-flight levels (*P* < 0.05) [29]. Hence, a change in microbial composition after traveling to space does not imply a loss of microbial species per se.

Taken together, the current data on the microbiome of space travelers suggest no immediate and severe space-induced dysbiosis, although large areas of microbiome functionality remain poorly understood. The following sections attempt to identify space-related knowledge gaps and, if possible, provide recommendations for solutions and experiments.

### Microbiome baseline determination and pre-flight preparations

**Fig. 1:**
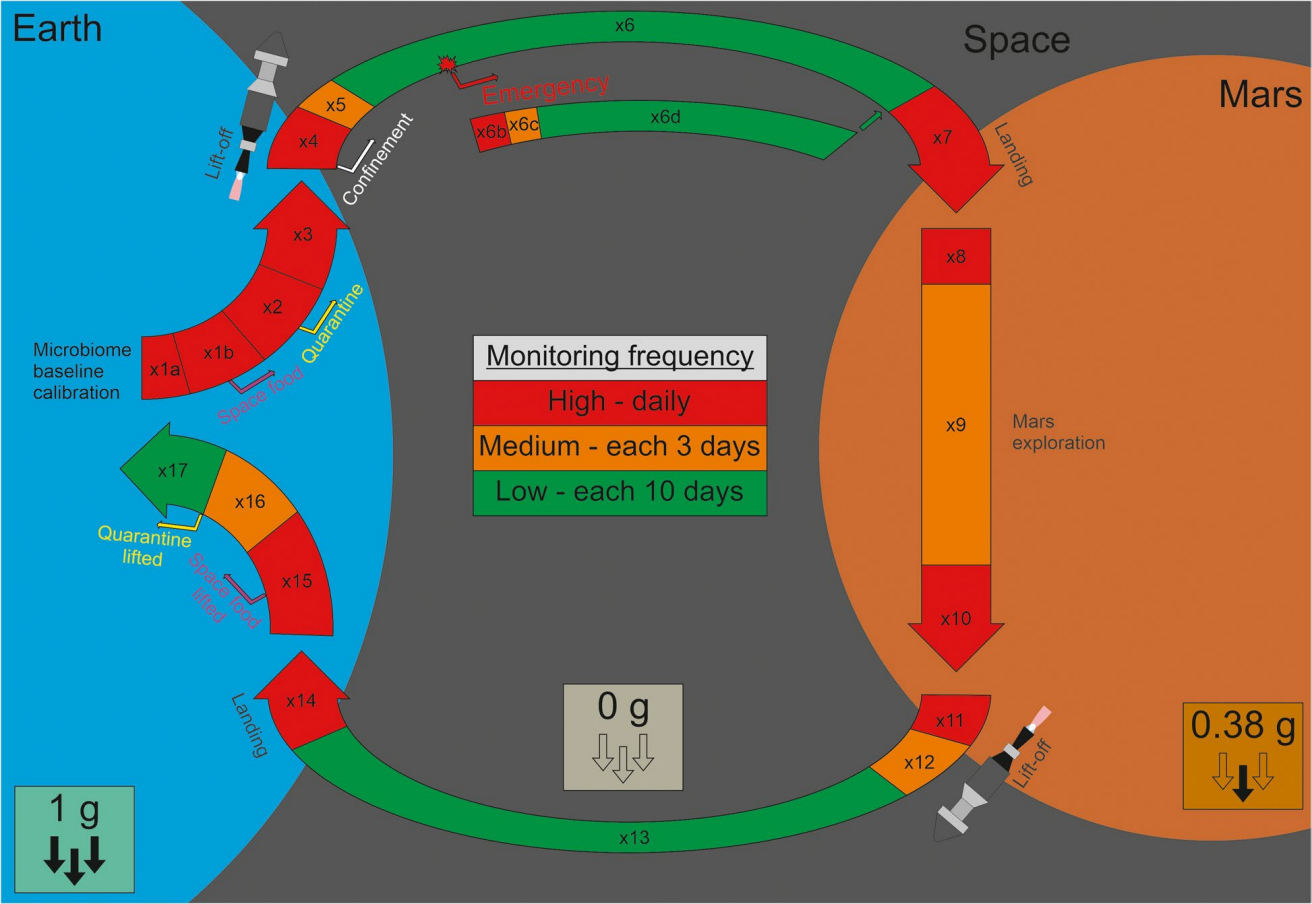
Schedule of proposed microbiome monitoring on the journey to Mars. Shown is the schedule for switching from regular food (x1a) to space food (x1b), mobility of the astronauts (free or quarantine – beginning in x2), gravity (Earth = 1 g, Mars = 0.38 g, and space is 0 g), microbiome monitoring frequency (high, medium and low), and monitoring adjustment in case of an emergency (emergency in x6 leading to x6b). Sampling frequency phases are numbered sequentially (x1–x17) throughout the process and contain the following rationale: maintaining a low sampling frequency during flight to and from Mars (x6, x6d, and x13); switching to medium frequency after familiarization on Mars (x9), after lift-off from Mars (x12) or after landing back on Earth (x16); and high-frequency phases shortly before and after landing on and lift-off from Mars (x7, x8, x10, x11), before returning to Earth (x14), the first month back on Earth (x15), or whenever there is an incident involving the astronauts’ gut microbiome (x6b)

Microbiome composition and fluctuations are highly individual. However, the knowledge about the healthy and diverse microbiome baseline and its taxonomic/functional fingerprint is an important reference for the entire mission in order to detect unusual perturbations and disease events. Thus, during *lift-off preparation*, the individual microbiome baseline of each space traveler should be determined while being in a state of good health, both by whole-metagenome sequencing (WMS) and targeted 16S rRNA gene amplicon sequencing covering all members (bacteria, archaea, eukaryotes, viruses/phages) of the microbiome. We suggest that the microbiome should be monitored daily at least for *14 days*, which is the duration of a perturbed microbiome then tending to reconstitute back to the initial state [10]. If perturbations to the microbiome occur during pre-flight monitoring, this increased frequency of observation could be extended until a steady-state microbiome is observed. Besides microbiome monitoring, meticulous recording of all *metadata* (food intake, health parameters, a stress diary, and more) will help to clarify scientifically important correlations [30–32]. This is particularly important, as the preoperational phase for lift-off might perturb the microbiome substantially by switching to a space diet, entering quarantine, or experiencing pre-lift-off stress (Fig. 1). These modifications should be introduced in phases, accompanied by daily microbiome monitoring. Only once these modifications have been monitored and fluctuations remain stable (with a new, adapted baseline being calibrated), lift-off should be considered.

Major threats to the space travelers’ health are *obligate pathogens*, possibly impairing the functionality of the crew members during flight and endangering the mission. Besides disease itself, obligate pathogens may lead to dysbiosis of the microbiome by causing gastrointestinal inflammation, including diarrhea, or forcing the space travelers to use antibiotics and provoking severe microbiome perturbations. By applying *strict sterilization* methods, obligate pathogens could be removed from any niche of the spacecraft microbiome (including the spacecraft itself, its cargo, surfaces, and devices) [33].

In order to avoid obligate or opportunistic pathogens concealed in the crew members’ microbiome, a strategy of *microbiome remodeling* could be followed, which is still to be researched and established in detail. Therein, the individual microbiome could be monitored, followed by targeted elimination of pathogens (by tools such as phage therapy or immunotherapy) and enrichment of beneficial strains (prebiotics, engineered probiotics) [34]. Attempts to remodel the microbiome, however, are still in a very early stage of development and require further fundamental research. The effects of eliminating a single, possibly predominant species in an established and stable microbiome are yet unknown. In addition to supplying space travelers with a beneficial microbiome, such techniques, if established and verified, would offer revolutionary possibilities in other fields of application on Earth.

### Type, length, and frequency of microbiome monitoring

As resources during space flight are expected to be limited**,** *monitoring frequencies* need to be adapted to balance the demands of health surveillance and the resources available. Considering these constraints, the authors reviewed studies reporting the sequence and duration of typical microbiome perturbations: starting from a diverse microbiome, a perturbation incident, a drop in richness, and reconstitution. Based on the findings, three categories of alert were defined allowing for the urgency of the situation. For example, David et al. monitored the microbiome daily during a *Salmonella* infection and showed that the perturbation lasted for 14 days [10]. The perturbation led to a shift in the steady state, as several species were lost while others increased in abundance. In particular, the change from the healthy pattern of diversity to the infected pattern of severely reduced diversity and abundance happened within three days. Therein, Proteobacteria (i.e., *Salmonella*) were observed and increased in abundance. After six days, the fluctuations of the microbiome slowly abated, reconstituting to a new steady state after 14 days.

Since the period between infection and perturbation was three days at its longest, any monitoring frequency should be shorter than this period. In light of this, during high alert levels, *daily* sampling and monitoring are recommended (Fig. 1, red arrows). During medium alert levels, a medium sampling frequency equal to the perturbation period of three days is recommended (Fig. 1, orange arrows). Finally, for the low alert periods, a longer cycle between the incubation period and the whole perturbation cycle of 10 days is recommended (Fig. 1, green arrows). Thus, an asymptomatic perturbation could probably still be identified. These frequencies are in accordance with other metagenomic studies, monitoring weekly in low-level alertness and daily on high-level alertness [35]. Outside of the fixed sampling schedule, in case of medical emergencies or illness, the monitoring frequency should be set to high before falling back to medium and then returning to low (Fig. 1, x6b, x6c, x6d).

Before traveling to Mars, this schedule should be reviewed and experimentally validated—for example, on the ISS or its successors.

During flight, monitoring frequencies of the gut microbiome should depend on the phases of the flight. High frequency with daily monitoring should be performed before, during, and after lift-off and landing from Earth and Mars, high and medium frequency during the presence on Mars (Fig. 1). Thereby, stress-induced perturbations and any Martian contamination might be quickly detected.

### Monitoring methodology, data analysis, and modeling

In order to monitor the microbiome, the use of *next-generation sequencing* (*NGS*) is the current gold standard. Whole-metagenome sequencing (WMS) allows broad-spectrum views of the microbiota at chosen time points for actual or post hoc analysis. The use of 16S rRNA gene amplicon sequencing enables the analysis of variations in known species for microbiome stability, dysbiosis detection, and crew health monitoring. It is the recommendation of this paper that the feasibility of performing both whole metagenome and 16S rRNA sequencing technology, while following established and commonly accepted metagenomic guidelines with an emphasis on a reduced bias [36], is investigated.

Microbiome monitoring on a space mission requires a portable, easy-to-use, resilient, and space-saving sequencing tool. Leggett et al. [37] used MinION by Oxford Nanopore Technologies to identify pathogenic bacteria and their corresponding antimicrobial resistance gene profiles within one hour of sequencing. Burton et al. [38] used MinION to identify microbes entirely in situ on the ISS, verifying its feasibility in space. Stahl-Rommel et al. [39] established a simplified method for real-time microbiota profiling onboard the ISS and made it accessible even for non-trained crew members.

With the space travelers’ microbiome permanently monitored, a system should be established translating sequence data into medical guidance and a warning system. Johnson et al. used microbiome baseline data to predict its composition the next day [13]. For health monitoring, such a model could be established for the crew members by incorporating it into an *automated warning system*. If the microbiome differed significantly from the previously predicted values, it could indicate an emerging perturbation. If sequencing depth manages to screen to species and subspecies levels, the pathogens could be identified directly. If a WMS approach is considered instead or additionally, pathogens could be detected by identifying genes involved in virulence. This warning system requires setting up a sequence database containing all known microorganisms including their functionality and beneficial or detrimental effects on human health, which could be based on standard databases like NCBInr, GTDB [40], eggNOG [41, 42], SEED [43], KEGG [44, 45], MetaCyc [46], VFDB [47, 48], EC [49], or InterPro2GO [50]. The warning system database will require further personalization for each crew member, using relevant sequences obtained from their pre-lift-off microbiome samples.

### Prevention of microbiome perturbations during long-term spaceflight

*Perturbations* should be prevented by prophylactic strategies, such as the reduction of pathogen load in the microbiome of the spacecraft and the crew, the latter by microbiome remodeling. The spacecraft and internal equipment should be *cleaned* to reduce the total microbial load, including screening afterwards to assess the subsequent microbial load [33].

In case of a severe infection during flight, *antibiotics* should be used only if absolutely necessary. As previously discussed, the crew are predicted to show a loss of species diversity over time, which should be monitored and, if observed, counteracted by long-term regular microbial input (i.e., by food) and reconstitution strategies (i.e., by autologous fecal microbiome transplant (aFMT) and others).

Methods of *stress reduction* should be established and consistently followed. In the Mars500 study, stress was a likely factor causing perturbation in the gut microbiome of the participants [23]. However, this study did not examine whether stress-induced perturbation led to a shift in the steady state or whether the microbiome was fully reconstituted after the stress ended. Further research needs to be undertaken to clarify this. In particular, it is the recommendation of this paper to (i) monitor the microbiome of healthy subjects who are about to encounter stressful situations; (ii) determine diversity fluctuations and any correlation between this and the subjective experience of stress; (iii) raise awareness for the space travelers and teach tools of stress management; (iv) in severe cases, prepare microbiome reconstitution; and finally, (v) investigate whether specific strains might be administered as probiotics which are known for the capacity to counteract stress signaling [51].

*Food design* (including types, preservation methods, preparing temperature, and freshness) directly influences the performance of a rich, diverse gut microbiome [52]. Currently, space travelers on the ISS are regularly supplied with preserved food from Earth. However, for long-term missions, food supply strategies have to be re-evaluated.

Food taken along can be *sterilized* with no viable microorganisms inside or conserved with low microbial abundance in a dormant stage. Bio-regenerative food sources (e.g., the future exploration greenhouse (FEG) within the EDEN ISS project), if considered, contain the *greenhouse’s* environmental microbiome [53, 54]. Depending on the source and food preservation method, the microbial diversity varies strongly. All methods should be evaluated on whether there are beneficial inoculation effects on the crew’s microbiome diversity by food microorganisms, the permeability for potential pathogens, and the loss of beneficial molecules due to sterilization procedures. Bio-regenerative life support systems and growing the food in space may be the long-term choice for humankind, as it gives space travelers independence from Earth supply, especially if there is an emergency on Mars and the space travelers become stranded. However, this also means the introduction of a new green plant-related microbiome to monitor, which again has to be investigated. Several questions should be answered beforehand: What are the effects on the gut microbiome when space travelers live on sterilized food for years? Do we see a loss of species and richness over time? What happens when eating food with microorganisms barely surviving the preservation process? What happens when space travelers eat self-grown food inoculated with the greenhouse microbiome? Can or should we supply food with individually chosen probiotics?

*Dietary fibers* are known to support SCFA-producing bacteria, which support microbial tolerance and health [55]. How and to what degree do dietary fibers (prebiotics) support the microbiome and what kind of food can be supplemented or grown to maintain a healthy level throughout the Mars mission?

*Loss of species*: When astronauts shared the same new environment (in the Mars500 experiment or on the ISS) containing its distinct and dynamic microbiome, the newly arrived crew members and their microbiomes were exposed to significant changes to the microbial influx as well as the overall environmental conditions. This led to a microbial rearrangement in the gut with low abundance microorganisms becoming more similar between the crew members (longitudinal homogenization) and a preservation of the dominant ones [11, 28]. After a rapid period of adaption (seven days), the fluctuations reached a new steady state, which persisted for at least six months in space [28].

For a long-term space mission, the conditions will appreciably differ from ISS protocols (three-fold extended duration, no cargo supply from Earth, no crew exchange during that time). The environment of the space travelers and thus its effects on their microbiome composition will change in small steps, starting from a microbiome baseline of each individual on Earth, switching to an increased presence in space agencies’ facilities and food, intensive training, and contact (possibly with other crew members), quarantine, space food, stress factors, lift-off preparation, lift-off, and alignment on the spacecraft. Each environmental change may alter the fine balance of microbial influx possibly affecting the equilibrium and, in the worst case, lead to a perturbation.

Once in space and acclimatized, the *unperturbed equilibrium* of the crew’s microbiome is affected by three factors: (1) a constant microbial influx deriving from the surrounding crew members, surface microbiomes, and food; (2) adherence properties and resilience of the gut microbiota; and (3) a possible loss of microorganisms, driven by detaching mucus, liquid flow, and gut peristalsis. The last of these may be a constant factor for loss of species over time. Even though ISS data suggests no significant reduction within six months, it must be elucidated if there might be a turning point where exchange with the new environment on the spacecraft and the other crew members is saturated with richness reaching a peak and possibly dropping again. Additionally, ISS members were exchanged every six months, with new crew members entering the ISS in between and possibly introducing new sets of microorganisms. In Mars500, the simulated spacecraft was on Earth and in contact with the staff. Although confined, it cannot be ruled out that these influxes had significant contributions to maintaining microbial diversity. It should be studied if by stringent isolation and the fact that no new species enter the environment accidently, the threshold of a microbial influx into the steady state might be undercut, initiating a constant loss of species over longer time periods.

A single round of treatment with *antibiotics* acts as a severe perturbation reducing microbial diversity. The effects on the healthy gut microbiome can be detected for months to years, as revealed in several studies [2, 3, 15, 56, 57]. Lost species were not recovered suggesting a shift towards a new equilibrium. Studies have shown that antibiotic treatment caused alterations to the microbiome that remained detectable for years after exposure [2, 15]. Likewise, treatment with antibiotics within the previous year is associated with a significantly increased likelihood of dysbiosis-associated diseases [14]. This pattern of treatment leading to dysbiosis-associated diseases can thereby become a vicious circle. Even though antibiotics are still an essential part of the medical treatment regime and should be part of the manifest on the voyage to Mars, their use should be restricted more than on Earth and the severe, long-term side-effects on the microbiome should be considered in the prescription approach alongside access to microbiome reconstitution strategies.

### Microbiome monitoring of spacecraft environment

Overall, it might be necessary to analyze in more detail the microbial community of different areas and *surfaces within spacecraft*/spacecraft mock-ups with respect to diversity, structure, quantity and distribution pattern, temporal fluctuations, and drifts to new steady states. This includes the detection of biofilms, toxins, and/or particle-associated microorganisms. The results should be systematically compared to suitable ground controls in order to estimate the impact of space and spaceflight conditions.

In one recent study, the ISS surface microbiome was found to be unique and different to the microbiomes of the ISS dust and HEPA filters, NASA's Jet Propulsion Laboratory cleanrooms, and Lunar/Mars analog habitats [58]. However, the ISS surface microbiome was similar to the Japanese module of the ISS and built environments on Earth frequented by humans (fitness centers, places of work, hospitals) [59]. This indicates that humans and their skin microbiomes shape the indoor microbiome, both on Earth and in space. These microorganisms may also live and survive on spacesuits used for spacecraft maintenance. In simulations on Earth, microorganisms were identified on space suits [60]. Future studies need to establish possible contamination routes from inside the spacecraft via space suits onto the exterior of the spacecraft, especially in terms of planetary protection of Mars.

Many of the organisms detected in the ISS surface microbiome were opportunistic pathogens and biofilm formers. *Biofilms*, in particular, should be a focus to address the concerns over bio-fouling, material degradation, and the effects on human health as well as to identify potential hot spots for risk estimation. Microbial contamination routes should be tracked, along with models to predict the distribution of microorganisms in confined habitats as a function of geometry, airflow, relative humidity, electric charge, surface materials, and temperature.

It is important to improve and standardize *environmental microbiology monitoring* for air, surfaces, water, and food during spaceflight in order to determine the microbial risk limits for future mission scenarios. In addition, the optimal type and frequency of microbial monitoring needs to be established. As further research widens our understanding of both the surface and gut microbiome, determining indicator microorganisms and thresholds for habitat stability should be undertaken if possible.

The authors recommend experimentally determining the effects of changing from the current stringent biocidal cleaning regime to a routine biostatic cleaning regime and a biocidal regime only during outbreaks of infection [61]. The biostatic regime may help maintain a suitably low biomass but diverse surface microbiome thus enhancing diversity and resilience in the crew’s microbiomes. A similar change from a biocidal to a biostatic disinfection regime in the Mars500 simulation did not result in observable dysbiosis in the simulated crew [24].

Hygiene products in general (including personal care products and anthropogenic chemicals) were shown to impact the microbiome on human skin [62] and the built environment [63]. Bouslimani et al. identified a person-, site-, and product-specific long-lasting response of molecular and bacterial diversity on the skin of 11 volunteers, while Hu and Hartmann emphasized the phenomenon of water availability, which determines the magnitude of how far microbes are affected by anthropogenic chemicals. These observations could have numerous implications for the long-term stays of humans in space: First of all, strict water restrictions might anyhow limit a wide choice of hygiene products. However, person-specific effects of personal care products also open the possibility to design a crew-specific precision skin care approach with beneficial effects on its microbiome. Secondly, while water availability is restricted in space, zero gravity results in the release of sweat droplets and condensation inside the spacecraft—an effect often visible by fungal growth onboard the ISS. Hence, in comparison with the terrestrial human habitats, water availability on surfaces inside a manned spacecraft might be higher, allowing microbes to use anthropogenic chemicals as carbon source for microbial growth. Therefore, detergents and antimicrobial cleansers should be selected with care to also avoid an increase of antibiotic resistances during space travel.

### Analysis of space effects on the microbiome

In the context of microbiology, space effects are a blanket term herein for changes in microbial activity when they are exposed to the environmental conditions of a space habitat or space itself, rather than a terrestrial environment (e.g., microgravity, cosmic radiation).

One of the space effects is constant high-energy *radiation* with intermittent higher bursts of high-energy solar particle events (SPE) from coronal mass ejections or solar flares [19, 64]. Without shielding, the background intensity of radiation in space is fatal for humans and highly mutagenic for microorganisms [64]. How would non-fatal doses of radiation alter the microbiome, its diversity, and virulence? What effect does cosmic radiation have on the crew and in particular on microorganisms beyond the Van Allen Radiation Belt? Experiences from radiotherapy for cancer treatment showed that radiation may remodel the microbiome composition and cause radiation enteritis, as reviewed by Liu et al. [65]. In a mouse model, long-term low-dose ionizing radiation (in the context of a deep space simulation with 0.1 to 1 Gy) increased the relative abundance of opportunistic pathogens [66].

How could space effects affect the human host and its immune system, and in further consequence, the interaction of host and microbiome? In a review on the effects of spaceflight on the *human microbiome and immune system*, Cervantes and Hong reported a variety of immunological changes such as reduced leukocyte counts, cytokine production, and CD3^+^ and dendritic cell activation [67]. In addition, the precise effects seemed to vary with the duration of the spaceflight. They further noted that a wide range of gastrointestinal bacteria demonstrated increased virulence features. For example, *Salmonella typhimurium* showed increased survival in macrophages and biofilm formation, *Escherichia coli* demonstrated greater adherence to gastrointestinal epithelial cell lines in culture, and increasing production of heat-labile enterotoxin and *Pseudomonas aeruginosa* also showed increased biofilm production [67].

Can we identify a *selection pressure* for microorganisms and/or whole microbiomes in space to a specific (possibly more virulent) situation? Indications for increased biofilm formation and adaptations with respect to surface attachment were frequently reported [59, 68].

What effect does *microgravity* have on the crew, on microorganisms, and on biochemistry in general? Microgravity does lead to a lack of convection in fluids, which in further consequence alters microbial behavior of sessile microorganisms, possibly due to reduced phosphate and/or oxygen availability [69]. Huang et al. concluded that despite 50 years of research into the effects of microgravity on the response of microbes to microgravity, most experiments focus mainly on growth rate and metabolism [70]. Furthermore, they revealed conflicting results, leaving the area of research inconclusive. The main cause of the discrepancy is due to differing strain selection and experimental conditions especially microgravity, cell motility properties, culture methods, and nutrient concentration of media [70].

### Microbiome reconstitution possibilities during space flight

In the confined habitat of a spacecraft or colony on Mars, the only sources for external microbiome *reconstitution* are other crew members, the habitat’s surfaces, air circulation and the water, and the food supply (whether from preserved food or from a greenhouse).

This is in contrast to the reconstitution of a microbiome on Earth where it has access to the infinite microbial diversity of our planet, including wide interpersonal contact, food, surfaces, soil, air, water, and animals. The effects of a reduced microbial diversity, due to strictly confined habitats, on the equilibrium of the human microbiome over longer time frames remain to be fully elucidated. If severe perturbations reduce the space travelers’ microbiome diversity during the mission, the diversity of the local habitat microbiome and any influx to the crew’s microbiome might not be sufficient to assure healthy reconstitution of the latter. In this case, backup strategies for reconstitution have to be established and made available.

In a study by Suez et al., it was shown that after antibiotic treatment *autologous (self) fecal microbiota transplantation* (*aFMT*) could reconstitute the microbiome within days of administration [71]. Similar results were found in a study comparing 14 aFMT-treated patients to 11 controls [72]. In both studies, the stool transfer after antibiotic treatment quickly reconstituted the microbiome to pre-intervention diversity levels [71, 72]. Under long-term isolated spacecraft conditions, possessing individual stool samples for emergency aFMT seems to be a viable option to reconstitute the microbiome in case of any perturbation. As preparation, such samples could be collected in the pre-flight phases when calibrating the baseline microbiome. However, further research covering the following questions will be necessary: (i) How can the aFMT procedure be conducted in a spacecraft environment, (ii) what storage options are available in a spacecraft and habitat on Mars for fecal microbiome samples (e.g., lyophilization, deep-freezing at − 80 °C or simply leaving it outside the spacecraft in sun-protected vacuum), and (iii) which protocol leads to the best preservation and reconstitution of the microorganisms during and after storage?

Even though the general public links *probiotics* to beneficial effects, scientific evidence is still scarce and the potential mechanisms should be further investigated [73, 74]. The healthy microbiome, to the extent we currently understand it, primarily consists of a set of species each individual human host learned to tolerate during their early years [6, 7]. Probiotics not incorporated into an individual’s tolerated set of microflora were found to have detrimental health outcomes as they delayed the reconstitution of the gut microbiome after antibiotic treatment in eight healthy volunteers [71]. These findings suggest that different people, with different microbiomes, respond differently to discrete probiotic strains and so, a presumed probiotic might be beneficial for person A, but not for person B. Any beneficial effects that are to be found from the use of probiotics most likely require an *individual approach*. If probiotics are considered as a space travel food supplement, a personalized in-flight probiotics administration protocol should be established during the pre-flight stages. This will require conducting research that (i) screens for microorganisms with known molecular mechanisms that improve human health (i.e., vitamin synthesis, SCFA synthesis) and establish a probiotics library, (ii) make sure no pathogenicity inducing genetic factors (e.g., virulence plasmids, pathogenicity islands) are present in the microorganisms’ genome, and (iii) screens the crew for immunological tolerance against the probiotics. Furthermore, it should be investigated whether it is possible to (iv) administer probiotics with minimal risk of immunological responsiveness to determine whether they can persist in the gut and produce the predicted beneficial effects and to (v) supplement any probiotics with prebiotics (such as dietary fiber) to strengthen the effects of either component. If all of these steps suggest the use of probiotics is desirable, additional research to determine the feasibility of building a viable probiotics library for the spacecraft should be undertaken.

As an alternative, or addition to aFMT, *heterologous transfer* of the GI microbiome could be considered between crew members. Even though there have been successful clinical treatments of chronic diarrhea with heterologous FMTs, in some cases FMT fails, for example, reviewed recently by Basson et al. [75] and Madoff et al. [76]. Additionally, a microbiome still contains a large spectrum of microorganisms, which are not necessarily safe for every individual. Therefore, safety concerns have to be eliminated pre-flight. It should be investigated whether it is possible to predict the compatibility between crew members, for example, by preceding metagenomics analysis of the crews’ microbiome and giving out a compatibility score [77]. Individuals with similar microbiomes might be more compatible.

Extraterrestrial experiments might require the presence of animals, such as insects, rodents, or higher mammals. Independent food strategies possibly depend on animals, microbial cultures, and/or plants. Pets and plants may be important for mental health and the moral of the crew. All of these would carry their individual microbiomes and add another layer of complexity to the diversity and reconstitution capacity of the crew [78–80].

### Human spaceflight in the context of planetary protection and human return to Earth

At the end of a mission to Mars, several new features have to be considered as the crew members, spacecraft, and samples return to Earth: (i) protection of the terrestrial ecosystem from possible Martian life; (ii) safely reintroducing the crew’s microbiomes to the general Earth microbiome, for their health and to ensure they do not transport slow-acting pathogens back to the terrestrial environment; and (iii) storing the microbiome data and samples to generate a resource of unparalleled value for subsequent experiments.

Before lift-off from Earth and before landing back on Earth, *all surfaces and crew* of the spacecraft should be screened in as much detail as possible, for example, by WMS. Creating a detailed catalog of most microorganisms at the start and end of the mission will enable the detection of space-related mutations and adaptions. If Martian life, extinct or extant, contains nucleotides with a similar base structure to those on Earth, WMS might be able to detect the presence of such life if unknown or exotic signals appear in the sequencing data. In that case, strict quarantine should be imposed until planetary protection is ensured.

A human mission to Mars will be the first long-term space mission, proposed lunar bases may produce data about long-term living outside LEO earlier. As such, it will generate invaluable data for the scientific community and planning future space missions. To prepare microbiome health surveillance strategies for future space missions, it is necessary to plan how to obtain as much clear data as possible. For example, considering the storage of stool and other microbiome samples at different time points throughout the mission so that extensive analysis can be conducted after the mission returns to Earth, including analyses developed after the mission parameters are set and the flight has departed.

Finally, what is the consequence for a microbiome that has been in the restricted environment of a mission to Mars when it returns to Earth? Turroni et al. showed that after leaving the Mars500 facilities, some of the participants returned to their pre-mission microbiome steady state and some changed to a new equilibrium [11]. It should be investigated whether these altered steady states have health implications for the individuals.

## Conclusions and research roadmap

The scientific achievements of humankind in the last 50 years are remarkable and incomparable to any past era. Humans have left planet Earth, lived in microgravity, sent space probes to as well as on other planets and into interstellar space, and set foot on the Moon. For comparison, the Apollo 11 mission (landing on the Moon and returning to Earth) took a total of *eight days*. The crewed journey to Mars, including landing and safely returning to Earth, will take months and years and will require a strenuous effort on a completely new level.

Microbiome awareness, at first glance, may be one of many little cogs in the big wheel, but neglecting this aspect can have severe implications for the space travelers’ health, and at its worst jeopardize the entire mission. The consequences of a fatal incident to Mars might set back space travel for generations.

As described in this commentary, studies are starting to emerge which will shed light on these complex microbiological questions (Mars500, HI-SEAS, on the ISS, and others), each one giving important insights to the bigger picture. Space agencies all over the world established roadmaps on solving problems arising from space-related microbiome alteration, such as NASA’s Human Research Roadmap [81] or ESA’s Roadmap for Future Research [82]. Nevertheless, we do not know enough yet to safeguard the crews’ health and functionality on such an extraordinary endeavor.

This commentary states important *knowledge gaps* of our understanding of a healthy space traveler’s microbiome and its maintenance and proper monitoring in space and gives specific recommendations how these gaps might be filled. Specifically, all preoperational experiments should follow *common rules* about accessible data management, metadata provision, and follow similar metagenomic protocols to make it easy for scientists all over the world to contribute and work together. The microbiome of space travelers should be monitored according to the given schedule with *NGS* technology (Fig. 1), e.g., a daily stool sampling frequency in critical mission stages. A computer-based system should be established, processing the sequence data for a timely *warning system*. Therein, microbiome dysbiosis could be detected and precise medical treatment targeting only the causative agent could be envisaged. Establishing such a system, on the long run, will also contribute to better medical treatment on Earth. All imaginable *perturbations* have to be simulated. These include known factors such as pathogen infections, antibiotics, stress, reduced environmental diversity, loss of species over time in confinement, and—most importantly—also the unknown factors of long-term exposure to space effects (particularly cosmic radiation and zero or reduced gravity). Strategies have to be established to prevent perturbations prophylactically (intensive spacecraft sterilization, food conservation, preparatory quarantine) and to give the crew the tools to cope with it on their journey (stress management, psychological support, antibiotic alternatives). Importantly, without access to the microbiota on Earth, *reconstitution* of the microbiome requires a pool of high microbial diversity and could be facilitated by aFMT, hFMT, individual probiotics, prebiotics, and food as well as greenhouse microbiota. For spacecraft integrity and planetary protection, the composition and diversity of the complete spacecraft microbiome have to be further investigated beforehand. Terrestrial extremophiles taken along might contaminate Mars, and biofilm-associated microorganisms might degrade spacecraft materials. In addition to the initial screening, the spacecraft microbiome has to be profiled before returning to Earth. The difference will reveal mutations and space adaptions along the way, and possibly indicate Martian stowaways. A Martian contamination and dissemination on Earth must be averted under all circumstances. The mission will harbor invaluable scientific knowledge for humankind. This treasure can be amplified if proper measures will be taken beforehand, for example, storing stool samples along the way. This gives future scientists the option to do experiments with it on Earth by not yet developed technologies.

For sure as we answer these questions, further questions will emerge. Some of these *untouched aspects* cover potential changed functioning of the immune system, metabolic capabilities, resistance and virulence of microbes in and on the space traveler’s body, the habitat, but also an enclosed life supporting system and its green plants, soil, water, and air, or approval of and limited shelf life of drugs and expected efficacy of medication in space.

Since we are aiming for a crewed journey to Mars as early as the 2030s, the next steps need to be taken in a *timely manner*.

## Data Availability

Not applicable

## Abbreviations

ISS: International Space Station
SCFA: Short-chain fatty acids
NGS: Next-generation sequencing
HI-SEAS: Hawaii Space Exploration Analog and Simulation IV
GIT: Gastrointestinal tract
LEO: Low Earth orbit
WMS: Whole-metagenome sequencing
aFMT: Autologous fecal microbiota transplantation
hFMT: Heterologous fecal microbiota transplantation
HEPA: Filter high-efficiency particulate air filter
SPE: Solar particle events
NASA: National Aeronautics and Space Administration
ESA: European Space Agency
